# Supplementary material for: Expanding the bat toolbox: Carollia perspicillata bat cell lines and reagents enable the characterization of viral susceptibility and innate immune responses
Source: PLoS Biol. 2025 Apr 15;23(4):e3003098. doi: 10.1371/journal.pbio.3003098 (PMC11999112; doi:10.1371/journal.pbio.3003098)
Supplement: S1 Table — Databases include the American Type Culture Collection (ATCC; USA), Collection of Cell Lines in Veterinary Medicine (CCLV; Germany), and Kunming Cell Bank of Chinese Academy of Science (KCB; China). References are included for cell lines not available through a database. (DOCX) [file pbio.3003098.s001.docx]

**Supplemental Table 1.** Cell types established from different bat species. Databases include the American Type Culture Collection (ATCC; USA), Collection of Cell Lines in Veterinary Medicine (CCLV; Germany), and Kunming Cell Bank of Chinese Academy of Science (KCB; China). References are included for cell lines not available through a database.

| Species | Tissue | Primary or Immortalized | Immortalization Technique | Cell Line | Commercial Source / Database | Ref. |
| --- | --- | --- | --- | --- | --- | --- |
| *Artibeus jamaicensis* | Embryonic liver | Primary |  | AjLi |  | [1] |
|  | Embryonic kidney | Primary |  | AjLKi |  | [2] |
|  | Embryonic intestine | Primary |  | AjIn |  | [2] |
|  | Kidney | Immortalized | SV40T | AJi |  | [3] |
|  | Intestine | Primary |  | Intestinal Organoids |  | [4] |
| *Aselliscus stoliczkanus* | Skin |  |  | STB-S2 | KCB  #5301MAM-KCB05010ZJ |  |
| *Carollia perspicillata* | Trachea | Immortalized | SV40T | CarperAEC.B |  | [5] |
|  | Lung | Primary |  | CpLu |  | [6] |
|  | Kidney | Immortalized | SV40T | CarNi/1 |  | [7] |
|  | Trachea | Primary |  | Airway Organoids |  | [8] |
|  | Lung | Primary |  | Airway Organoids |  | [8] |
| *Cynopterus sphinx* | Lung |  |  | GsnFB-L2 | KCB  #5301MAM-KCB0502LZJ |  |
|  | Skin |  |  | GsnFB-S1 | KCB  #5301MAM-KCB0501SZJ |  |
| *Desmodus rotundus* | Embryonic liver | Primary |  | DsLi |  | [1] |
|  | Embryonic kidney | Primary |  | DsKi |  | [1] |
|  | Embryonic lung | Immortalized | SV40T | FLuDero |  | [9] |
| *Eidolon helvum* | Embryonic kidney | Immortalized | SV40T | EidNi/41 |  | [10] |
|  | Trachea | Immortalized | SV40T | EidheAEC.B |  | [5] |
|  | Lung | Immortalized | SV40T | EidLu/20 |  | [10, 11] |
|  | Lung | Immortalized | SV40T | EidLu/43 |  | [10, 12] |
|  | Kidney | Immortalized | SV40T | ZFBS13-75A |  | [13] |
|  | Kidney | Immortalized | SV40T | ZFBK13-76E |  | [14] |
| *Eonycteris spelaea* | Lung |  |  | DB-L1 | KCB  #5301MAM-KCB0683LZJ |  |
|  | Muscle |  |  | DB-M1 | KCB  #5301MAM-KCB0683MZJ |  |
|  | Trachea | Primary |  | Airway Organoids |  | [15] |
| *Epomops buettikoferi* | Kidney | Immortalized | SV40T | EpoNi/22 |  | [16] |
| *Epomophorus gambianus* | Kidney | Immortalized | SV40T | ZFBK11-97 |  | [17] |
| *Eptesicus fuscus* | Kidney | Immortalized | MyPVT | Efk3 | Kerafast  #CVCL_GZ34 | [18] |
| *Eptesicus nilssonii* | Kidney | Primary |  | HAMOI-EnK |  | [19] |
| *Eptesicus serotinus* | Brain |  |  | FLG-ID | CCLV | [20] |
|  | Brain |  |  | FLG-R | CCLV | [20] |
|  | Kidney |  |  | FLN-R | CCLV | [20] |
| *Hipposideros abae* | Lung | Immortalized | SV40T | HipaLu/24 |  | [21] |
|  | Lung | Immortalized | SV40T | HipaLu/27 |  | [21] |
| *Hipposideros armiger* | Skin |  |  | GRB-S1 | KCB  #5301MAM-KCB04046ZJ |  |
| *Hipposideros caffer* | Embryonic | Immortalized | SV40T | HipaEm/5 |  | [21] |
|  | Embryonic | Immortalized | SV40T | HipEm/28 |  | [21] |
| *Hypsignathus monstrosus* | Embryonic kidney | Immortalized | SV40T | HypNi/1 |  | [16] |
|  | Kidney | Immortalized | SV40T | HypNi/21 |  | [21] |
|  | Lung | Immortalized | SV40T | HypLu/2 |  | [22] |
|  | Embryonic Lung | Immortalized | SV40T | HypLu/45 |  | [2] |
| *Macrotus waterhousii* | Heart |  |  | Mw1Ht | ATCC #CRL-6013  *discontinued* |  |
|  | Lung |  |  | Mw2Lu | ATCC #CRL-6014 *discontinued* |  |
| *Miniopterus fuliginosus* | Kidney | Immortalized | SV40T | YubFKT1 |  | [17] |
|  | Kidney | Immortalized | SV40T | YubFKT2 |  | [23] |
| *Molossus sinaloae* | Embryonic liver | Primary |  | MsLi |  | [1] |
|  | Embryonic kidney | Primary |  | MsKi |  | [1] |
|  | Embryonic intestine | Primary |  | MsIn |  | [1] |
| *Myotis altarium* | Skin |  |  | SM-S1 | KCB  #5301MAM-KCB04041ZJ |  |
| *Myotis daubentonii* | Brain | Immortalized | SV40T | MyDauBrain/48 |  | [21] |
|  | Intestine | Immortalized | SV40T | MyDauDa/46 |  | [21] |
|  | Lung | Immortalized | SV40T | MyDauLu/47 |  | [16] |
|  | Kidney |  |  | MyDauNi/2 |  | [7] |
| *Myotis davidii* | Kidney | Primary |  | MdKi |  | [24] |
| *Myotis myotis* | Brain | Immortalized | SV40T | MmBr |  | [25] |
|  | Tonsil | Immortalized | SV40T | MmTo |  | [25] |
|  | Peritoneal cavity | Immortalized | SV40T | MmPca |  | [25] |
|  | Nasal epithelium | Immortalized | SV40T | MmNep |  | [25] |
|  | Nervus olfactorius | Immortalized | SV40T | MmNol |  | [25] |
|  | Tail | Induced pluripotent stem cells |  | Mmy iPSCs |  | [26] |
|  | Skin |  |  | MMY-S2 | KCB  #5301MAM-KCB04044ZJ |  |
| *Myotis schreibersii* | Lymph node | Primary |  | MsLn |  | [27] |
|  | Kidney | Primary |  | MsKi |  | [27] |
|  | Kidney | Immortalized | SV40T | SuBK12-08 |  | [17] |
| *Myotis velifer* | Interscapular tumor | Cancerous |  | Mvi/It | ATCC #CRL-6012  *discontinued* | [28] |
|  | Muscle |  |  | Mvi/Mu | ATCC #CRL-6011  *discontinued* |  |
| *Pipistrellus ceylonicus* | Embryonic | Spontaneously Immortalized |  | NIV-BtEPC |  | [29] |
| *Pipistrellus nathusii* | Kidney | Primary |  | bKEC |  | [30] |
| *Pipistrellus pipistrellus* | Kidney | Immortalized | SV40T | PipNi/1 |  | [7] |
|  | Kidney | Immortalized | SV40T | PipNi/3 |  | [7] |
|  | Kidney | Immortalized | SV40T | PipNi/4 |  | [21] |
| *Pipistrellus subflavus* | Lung | Immortalized | hTERT | PESU-B5L |  | [31] |
| *Pteropus alecto* | Aorta | Primary |  | PaAo |  | [32] |
|  | Bone marrow | Primary |  | PaBm |  |  |
|  | Brain | Primary |  | PaBr |  |  |
|  |  | Immortalized | SV40T | PaBrT01-03 |  |  |
|  |  | Immortalized | hTERT | PaBrH01-07 |  |  |
|  | Fetus | Primary |  | PaFe |  |  |
|  |  | Immortalized | SV40T | PaFeT01-10 |  |  |
|  | Embryonic Membrane | Primary |  | PaFm |  |  |
|  | Heart | Primary |  | PaHe |  |  |
|  | Kidney | Primary |  | PaKi |  |  |
|  |  | Immortalized | SV40T | PaKiT01-03 |  |  |
|  | Liver | Primary |  | PaLi |  |  |
|  | Lymph node | Primary |  | PaLn |  |  |
|  | Lung | Primary |  | PaLu |  |  |
|  |  | Immortalized | SV40T | PaLuT01-04 |  |  |
|  | Muscle | Primary |  | PaMu |  |  |
|  | Pharynx | Primary |  | PaPh |  |  |
|  | Placenta | Primary |  | PaPl |  |  |
|  | Salivary gland | Primary |  | PaSg |  |  |
|  | Small intestine | Primary |  | PaSi |  |  |
|  | Skin | Primary |  | PaSk |  |  |
|  | Spleen | Primary |  | PaSp |  |  |
|  | Testes | Primary |  | PaTe |  |  |
|  | Thymus | Primary |  | PaTh |  |  |
|  | Uterus | Primary |  | PaUt |  |  |
| *Pteropus dasymallus yayeyamae* | Kidney | Immortalized | SV40T | FBKT1 |  | [33] |
| *Pteropus giganteus* | Spleen | Immortalized | SV40T | IndFSPT1 |  | [17] |
| *Pteropus pselaphon* | 5th finger of right wing and skin | Immortalized | CDK4, CYCLIN D1, and TERT | Bff-K4DT |  | [34] |
| *Rhinolophus affinis* | Fetus | Primary |  | BEF |  | [35] |
| *Rhinolophus alycone* | Lung | Immortalized | SV40T | RhiLu/1.1 |  | [6] |
|  | Kidney | Immortalized | SV40T | RhiNi/1.2 |  | [3, 21] |
|  | Brain | Immortalized | SV40T | RhiBrain/4p |  | [21] |
| *Rhinolophus euryale* | Lung | Immortalized | SV40T | RhiEuLu |  | [21] |
| *Rhinolophus ferrumequinum* | Kidney | Immortalized | SV40T | BKT1 |  | [17] |
|  | Lung | Immortalized | SV40T | RhiFeLu |  | [21] |
|  | Fetus | Induced pluripotent stem cells |  | Rfe iPSCs |  | [26] |
|  | Pulmonary | Immortalized | SV40T | RfPT |  | [36] |
|  | Brian | Immortalized | SV40T | RfBT |  |  |
|  | Heart | Immortalized | SV40T | RfHT |  |  |
|  | Kidney | Immortalized | SV40T | RfKT |  |  |
|  | Lung | Immortalized |  | GHB-L3 | KCB  #5301MAM-KCB0605LZJ |  |
|  | Ear skin |  |  | GHB-S3 | KCB  #5301MAM-KCB0605SZJ |  |
| *Rhinolophus landeri* | Lung | Immortalized | SV40T | RhiLu/1.1 |  | [7] |
|  | Kidney | Immortalized | SV40T | RIKd |  | [21] |
| *Rhinolophus lepidus* | Kidney | Spontaneously Immortalized |  | Rhileki |  | [37] |
| *Rhinolophus pusillus* | Lung |  |  | LHB-L2 | KCB  #5301MAM-KCB04052ZJ |  |
|  | Muscle |  |  | LHB-M4 | KCB  #5301MAM-KCB05058ZJ |  |
|  | Skin |  |  | LHB-S3 | KCB  #5301MAM-KCB05012ZJ |  |
| *Rhinolophus sinicus* | Kidney | Immortalized | SV40T | RsKT |  | [24] |
|  | Splenocytes | Primary |  | BS |  | [35] |
|  | Skin |  |  | CHB-S2 | KCB  #5301MAM-KCB05024ZJ |  |
|  | Intestine | Primary |  | Intestinal Organoids |  | [38] |
| *Rousettus ageyptiacus* | Kidney | Immortalized | SV40T | RoNi/7.1 |  | [16] |
|  | Body of fetus | Immortalized | Adenovirus | R06E |  | [39] |
|  | Head of fetus | Immortalized | Adenovirus | R05T |  | [39] |
|  | Vertebrate column of fetus | Immortalized | Adenovirus | R05R |  | [39] |
|  | Lung | Primary |  | RALU |  | [40] |
|  | Kidney | Primary |  | RaKSM |  | [40] |
|  | Kidney | Immortalized | SV40T | RaKSM-2.5i |  | [3] |
|  | Kidney | Immortalized | SV40T | ZFBK15-137RA |  | [23] |
|  | Endometrium | Immortalized | SV40T | RoEnd/4 |  | [21] |
|  | Lung | Primary |  | Airway Organoids |  | [41] |
|  | Intestine | Primary |  | Intestinal Organoids |  | [41] |
| *Rousettus leschenaulti* | Kidney | Immortalized | SV40T | DemKT1 |  | [17] |
|  | Intestine | Primary |  | Intestinal Organoids |  | [42] |
|  | Lung |  |  | FFB-L3 | KCB #5301MAM-KCB0520LZJ |  |
|  | Skin |  |  | FFB-S3 | KCB  #5301MAM-KCB0521SZJ |  |
| *Tadarida brasiliensis* | Lung | Primary |  | Tb1-Lu | ATCC #CCL-88 |  |

**SUPPLEMENTAL REFEREENCES**

1. Moreira-Soto, A., C. Soto-Garita, and E. Corrales-Aguilar, *Neotropical primary bat cell lines show restricted dengue virus replication.* Comparative Immunology, Microbiology and Infectious Diseases, 2017. **50**: p. 101-105.

2. Miller, M.R., et al., *Broad and temperature independent replication potential of filoviruses on cells derived from old and new world bat species.* The Journal of Infectious Diseases, 2016. **214**(suppl_3): p. S297-S302.

3. Letko, M., A. Marzi, and V. Munster, *Functional assessment of cell entry and receptor usage for SARS-CoV-2 and other lineage B betacoronaviruses.* Nature microbiology, 2020. **5**(4): p. 562-569.

4. Hashimi, M., et al., *Antiviral responses in a Jamaican fruit bat intestinal organoid model of SARS-CoV-2 infection.* Nature Communications, 2023. **14**(1): p. 6882.

5. Eckerle, I., et al., *Bat airway epithelial cells: a novel tool for the study of zoonotic viruses.* PloS one, 2014. **9**(1): p. e84679.

6. Hoffmann, M., et al., *Differential sensitivity of bat cells to infection by enveloped RNA viruses: coronaviruses, paramyxoviruses, filoviruses, and influenza viruses.* PloS one, 2013. **8**(8): p. e72942.

7. Müller, M.A., et al., *Human coronavirus EMC does not require the SARS-coronavirus receptor and maintains broad replicative capability in mammalian cell lines.* MBio, 2012. **3**(6): p. 10.1128/mbio. 00515-12.

8. Su, A., et al., *Infection Studies with Airway Organoids from Carollia perspicillata Indicate That the Respiratory Epithelium Is Not a Barrier for Interspecies Transmission of Influenza Viruses.* Microbiology spectrum, 2023. **11**(2): p. e03098-22.

9. Sarkis, S., et al., *Development of molecular and cellular tools to decipher the type I IFN pathway of the common vampire bat.* Developmental & Comparative Immunology, 2018. **81**: p. 1-7.

10. Biesold, S.E., et al., *Type I interferon reaction to viral infection in interferon-competent, immortalized cell lines from the African fruit bat Eidolon helvum.* PloS one, 2011. **6**(11): p. e28131.

11. Ng, M., et al., *Filovirus receptor NPC1 contributes to species-specific patterns of ebolavirus susceptibility in bats.* Elife, 2015. **4**: p. e11785.

12. Hoffmann, M., et al., *Tetherin inhibits Nipah virus but not Ebola virus replication in fruit bat cells.* Journal of virology, 2019. **93**(3): p. 10.1128/jvi. 01821-18.

13. Maruyama, J., et al., *Characterization of the glycoproteins of bat-derived influenza viruses.* Virology, 2016. **488**: p. 43-50.

14. Ogawa, H., et al., *Characterization of a novel bat adenovirus isolated from straw-colored fruit bat (Eidolon helvum).* Viruses, 2017. **9**(12): p. 371.

15. Chan, L.L., et al., *Generation of self-replicating airway organoids from the cave nectar bat Eonycteris spelaea as a model system for studying host–pathogen interactions in the bat airway epithelium.* Emerging Microbes & Infections, 2023. **12**(1): p. e2148561.

16. Kühl, A., et al., *Comparative analysis of Ebola virus glycoprotein interactions with human and bat cells.* The Journal of infectious diseases, 2011. **204**(suppl_3): p. S840-S849.

17. Maruyama, J., et al., *Characterization of the envelope glycoprotein of a novel filovirus, lloviu virus.* Journal of virology, 2014. **88**(1): p. 99-109.

18. Banerjee, A., et al., *Generation and Characterization of Eptesicus fuscus (Big brown bat) kidney cell lines immortalized using the Myotis polyomavirus large T-antigen.* Journal of virological methods, 2016. **237**: p. 166-173.

19. Horie, M., et al., *Establishment and characterization of a cell line derived from Eptesicus nilssonii.* Journal of Veterinary Medical Science, 2016. **78**(11): p. 1727-1729.

20. Richter, M., et al., *The viral envelope is not sufficient to transfer the unique broad cell tropism of Bungowannah virus to a related pestivirus.* Journal of General Virology, 2014. **95**(10): p. 2216-2222.

21. Gützkow, T., *Interspecies-transmission of animal coronaviruses*. 2013, Hannover, Tierärztliche Hochsch., Dis., 2013.

22. Krüger, N., et al., *Attachment protein G of an African bat henipavirus is differentially restricted in chiropteran and nonchiropteran cells.* Journal of virology, 2014. **88**(20): p. 11973-11980.

23. Sato, M., et al., *Generation of bat-derived influenza viruses and their reassortants.* Scientific reports, 2019. **9**(1): p. 1158.

24. Liang, Y.-Z., et al., *Cloning, expression, and antiviral activity of interferon β from the Chinese microbat, Myotis davidii.* Virologica Sinica, 2015. **30**: p. 425-432.

25. He, X., et al., *Establishment of Myotis myotis cell lines-model for investigation of host-pathogen interaction in a natural host for emerging viruses.* PLoS One, 2014. **9**(10): p. e109795.

26. Déjosez, M., et al., *Bat pluripotent stem cells reveal unusual entanglement between host and viruses.* Cell, 2023. **186**(5): p. 957-974. e28.

27. Zhang, H., et al., *A novel bat herpesvirus encodes homologues of major histocompatibility complex classes I and II, C-type lectin, and a unique family of immune-related genes.* Journal of virology, 2012. **86**(15): p. 8014-8030.

28. Shabman, R.S., et al., *Isolation and characterization of a novel gammaherpesvirus from a microbat cell line.* MSphere, 2016. **1**(1): p. 10.1128/msphere. 00070-15.

29. Mourya, D.T., et al., *Establishment of cell line from embryonic tissue of Pipistrellus ceylonicus bat species from India & its susceptibility to different viruses.* The Indian journal of medical research, 2013. **138**(2): p. 224.

30. Povolyaeva, O., et al., *Listeria monocytogenes infection of bat Pipistrellus nathusii epithelial cells depends on the invasion factors InlA and InlB.* Pathogens, 2020. **9**(11): p. 867.

31. Huynh, J., et al., *Evidence supporting a zoonotic origin of human coronavirus strain NL63.* Journal of virology, 2012. **86**(23): p. 12816-12825.

32. Crameri, G., et al., *Establishment, immortalisation and characterisation of pteropid bat cell lines.* PloS one, 2009. **4**(12): p. e8266.

33. Maeda, K., et al., *Isolation of novel adenovirus from fruit bat (Pteropus dasymallus yayeyamae).* Emerging infectious diseases, 2008. **14**(2): p. 347.

34. Tani, T., et al., *Establishment of immortalized primary cell from the critically endangered Bonin flying fox (Pteropus pselaphon).* PLoS One, 2019. **14**(8): p. e0221364.

35. Li, J., et al., *Molecular characterization of RIG-I, STAT-1 and IFN-beta in the horseshoe bat.* Gene, 2015. **561**(1): p. 115-123.

36. Geng, R., et al., *Unconventional IFNomega-like Genes Dominate the Type I IFN Locus and the Constitutive Antiviral Responses in Bats.* J Immunol, 2024. **213**(2): p. 204-213.

37. Auerswald, H., et al., *A Look inside the Replication Dynamics of SARS-CoV-2 in Blyth’s Horseshoe Bat (Rhinolophus lepidus) Kidney Cells.* Microbiology Spectrum, 2022. **10**(3): p. e00449-22.

38. Zhou, J., et al., *Infection of bat and human intestinal organoids by SARS-CoV-2.* Nature medicine, 2020. **26**(7): p. 1077-1083.

39. Jordan, I., et al., *Cell lines from the Egyptian fruit bat are permissive for modified vaccinia Ankara.* Virus research, 2009. **145**(1): p. 54-62.

40. Seifert, S.N., et al., *Rousettus aegyptiacus bats do not support productive Nipah virus replication.* The Journal of infectious diseases, 2020. **221**(Supplement_4): p. S407-S413.

41. Kellner, M., et al., *Reconstructing bat antiviral immunity using epithelial organoids.* biorrXiv, 2024.

42. Elbadawy, M., et al., *Establishment of intestinal organoid from rousettus leschenaultii and the susceptibility to bat-associated viruses, SARS-CoV-2 and pteropine orthoreovirus.* International Journal of Molecular Sciences, 2021. **22**(19): p. 10763.
