# Supplementary material for: Expanding the bat toolbox: Carollia perspicillata bat cell lines and reagents enable the characterization of viral susceptibility and innate immune responses
Source: PLoS Biol. 2025 Apr 15;23(4):e3003098. doi: 10.1371/journal.pbio.3003098 (PMC11999112; doi:10.1371/journal.pbio.3003098)
Supplement: S1 Fig — (A) Immortalized Artibeus jamaicensis (Aji) cells were infected with indicated pseudotypes at passages 8 and 25. (B) Immortalized Eptesicus fuscus (EfK3B) cells were infected with indicated pseudotypes. (C) Primary Rousettus aegyptiacus kidney and lung cells were infected at passage 1 with indicated pseudotypes. (D) R. aegyptiacus kidney cells transduced with SV40 T-antigen were infected with indicated pseudotypes. The data underlying this figure can be found in the S1 Data file available from the journal. (DOCX) [file pbio.3003098.s002.docx]

**
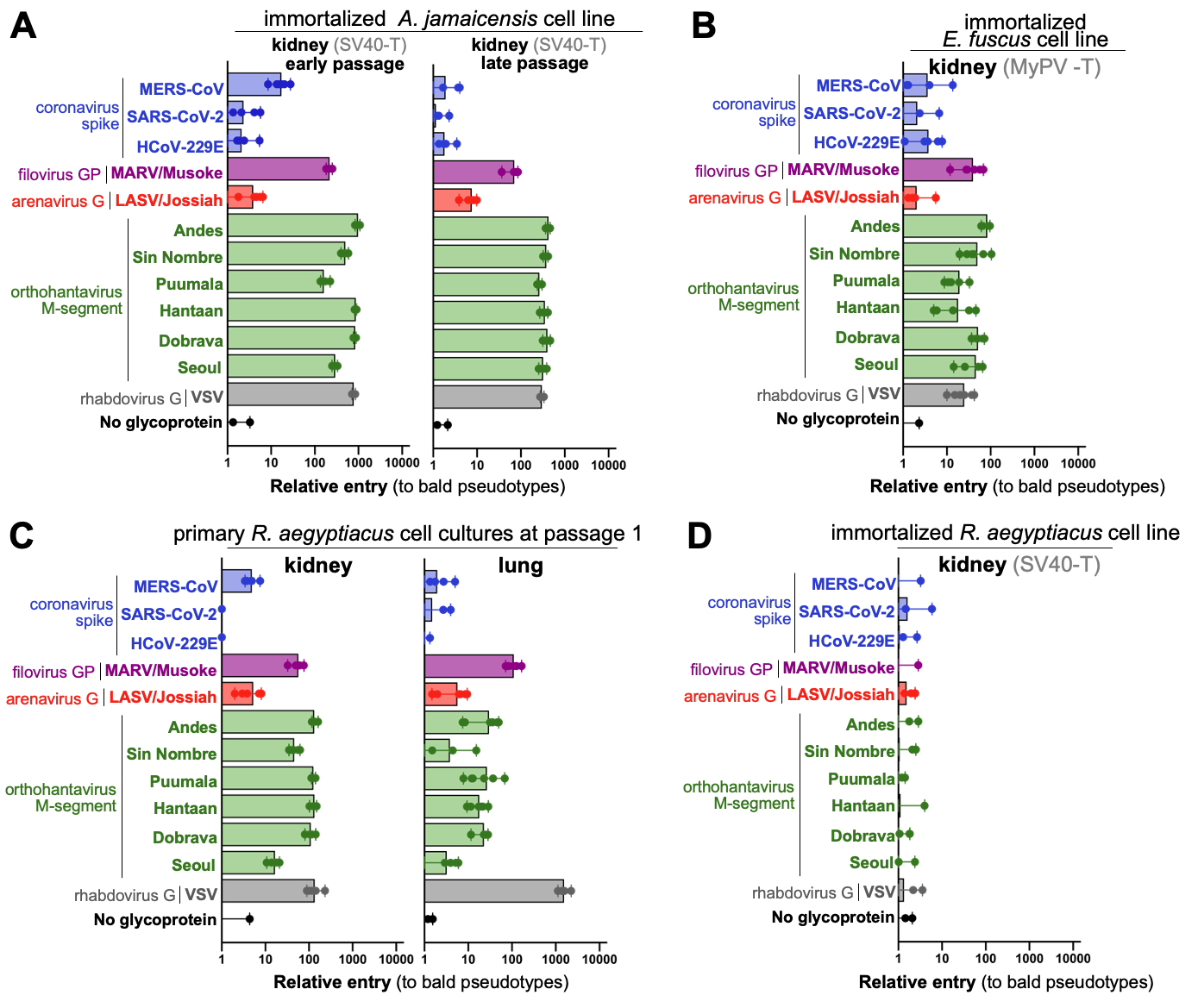
**

**Supplemental figure 1. Susceptibility of select previously published bat cell lines (A)** Immortalized *Artibeus jamaicensis* (AJi) cells were infected with indicated pseudotypes at passages 8 and 25. **(B)** Immortalized *Eptesicus fuscus* (EfK3B) cells were infected with indicated pseudotypes. **(C)** Primary *Rousettus aegyptiacus* kidney and lung cells were infected at passage 1 with indicated pseudotypes. **(D)** *Rousettus aegyptiacus* kidney cells transduced with SV40 T-antigen were infected with indicated pseudotypes.
