# Supplementary material for: Expanding the bat toolbox: Carollia perspicillata bat cell lines and reagents enable the characterization of viral susceptibility and innate immune responses
Source: PLoS Biol. 2025 Apr 15;23(4):e3003098. doi: 10.1371/journal.pbio.3003098 (PMC11999112; doi:10.1371/journal.pbio.3003098)
Supplement: S2 Fig — (A) Immortalized Carollia perspicillata cells were maintained in culture for multiple passages and infected with the indicated pseudotypes. (B) Late passage immortalized Carollia kidney cells were infected with indicated pseudotypes. All infections were performed in four replicates. The data underlying this figure can be found in the S1 Data file available from the journal. (DOCX) [file pbio.3003098.s003.docx]

**
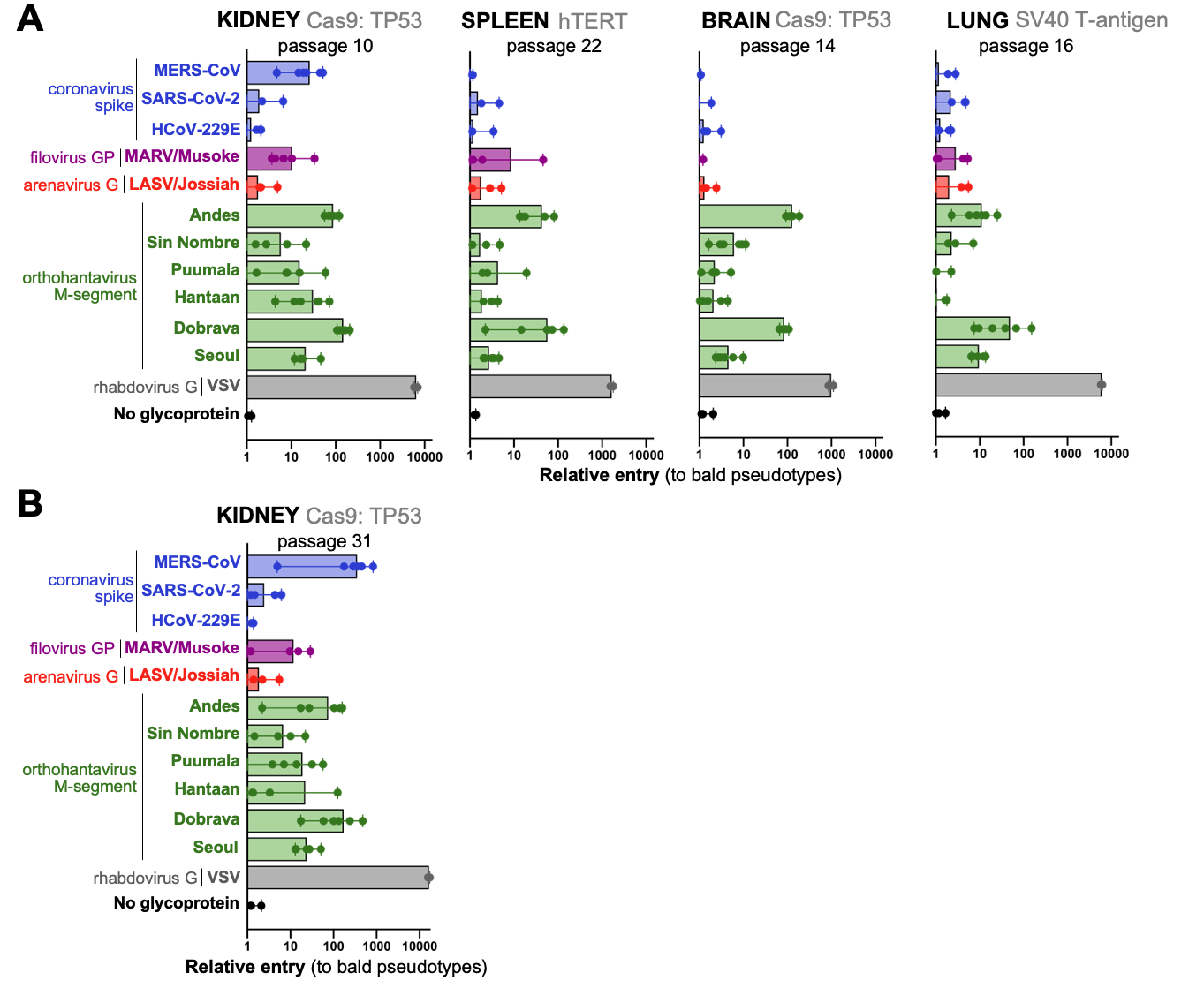
**

**Supplemental figure 2. Susceptibility of late-passage immortalized cultures. (A)** Immortalized *Carollia perspicillata* cells were maintained in culture for multiple passages and infected with the indicated pseudotypes. **(B)** Late passage oimmirtalized Carollia kidney cells were infected with indicated pseudotypes. All infections were performed in four replicates.
