# Supplementary material for: Expanding the bat toolbox: Carollia perspicillata bat cell lines and reagents enable the characterization of viral susceptibility and innate immune responses
Source: PLoS Biol. 2025 Apr 15;23(4):e3003098. doi: 10.1371/journal.pbio.3003098 (PMC11999112; doi:10.1371/journal.pbio.3003098)
Supplement: S3 Fig — (A) Primary kidney, spleen, brain, liver, and lung cell cultures. (B) Heterogenous cell populations are visible in the primary cell cultures. Images were acquired at 4× and 10× magnification on an EVOS M3000 microscope. (DOCX) [file pbio.3003098.s004.docx]

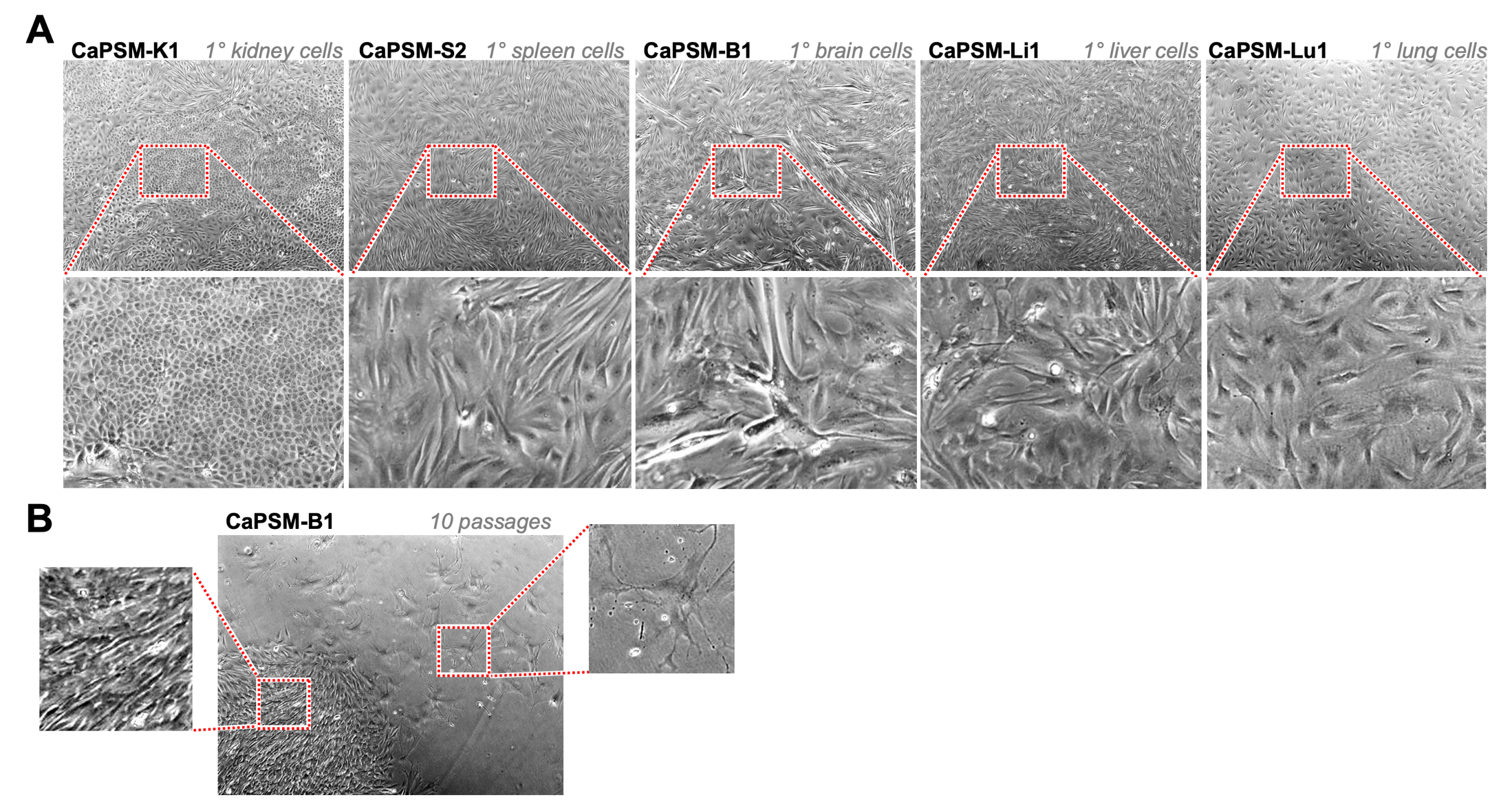


**Supplemental Figure 3. Bright field image of primary CaPsm cells**. (**A**) Primary kidney, spleen, brain, liver and lung cell cultures. (**B**) Heterogenous cell populations are visible in the primary cell cultures. Images was acquired at 4X and 10X magnification on an EVOS M3000 microscope.
