# Supplementary figures and images for: Expanding the bat toolbox: Carollia perspicillata bat cell lines and reagents enable the characterization of viral susceptibility and innate immune responses
Source: PLoS Biol. 2025 Apr 15;23(4):e3003098. doi: 10.1371/journal.pbio.3003098 (PMC11999112; doi:10.1371/journal.pbio.3003098)

Raw images for Figure 4E

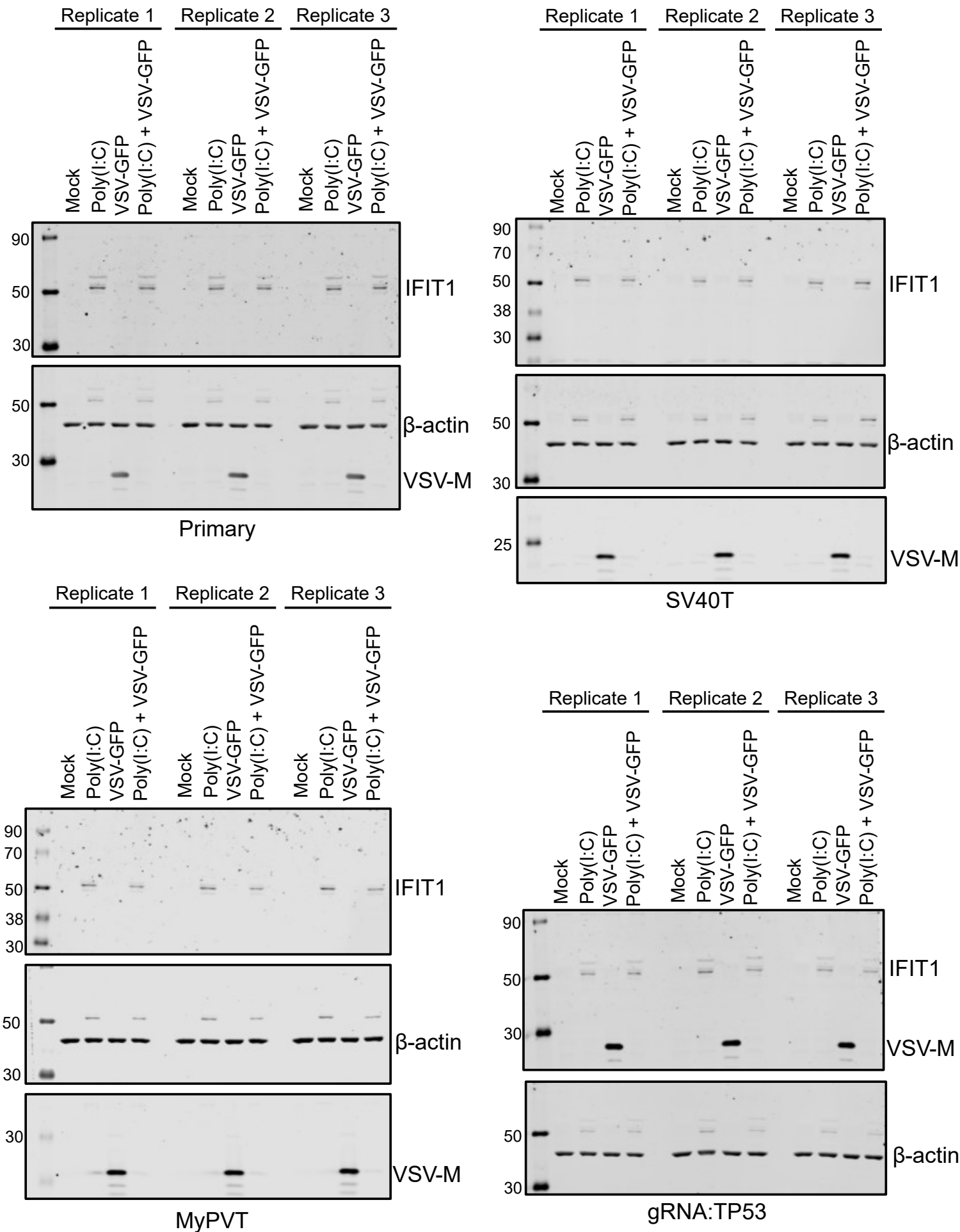

Raw images for Figure 4G

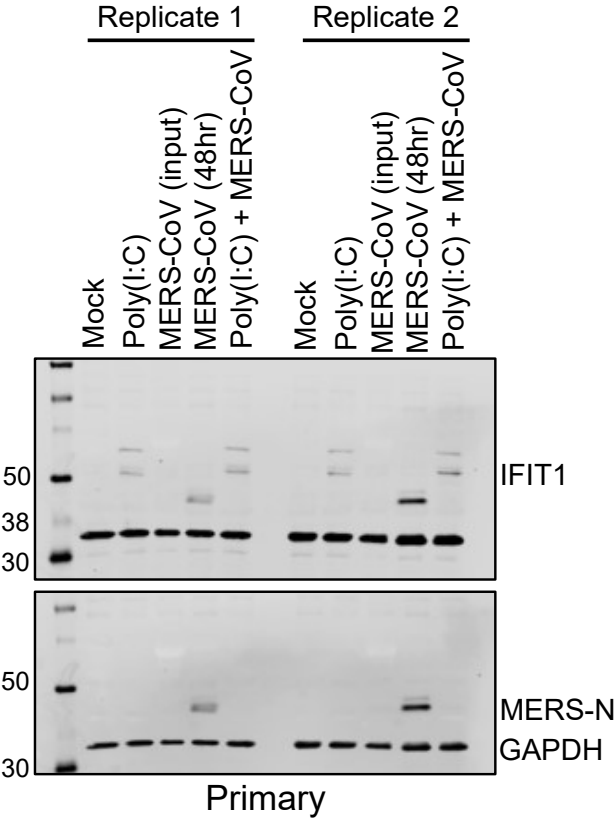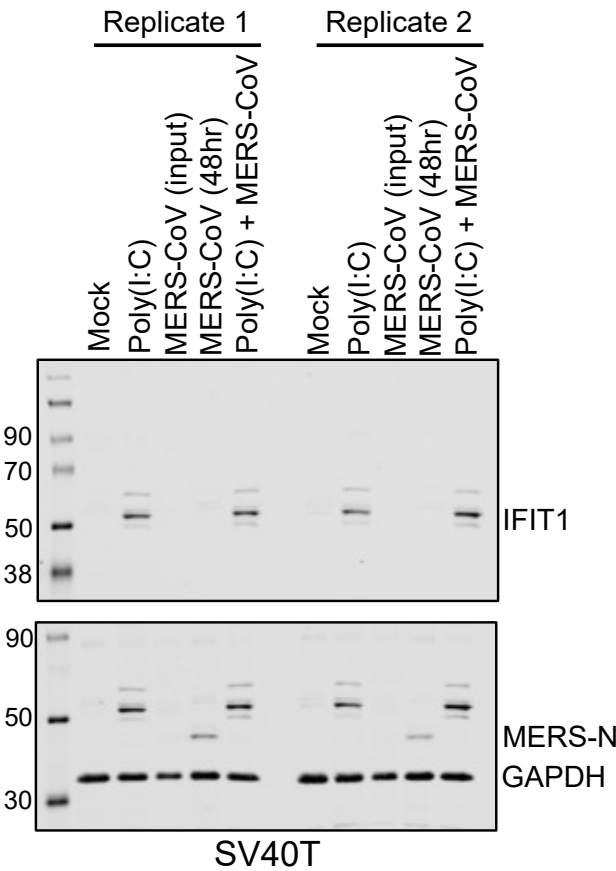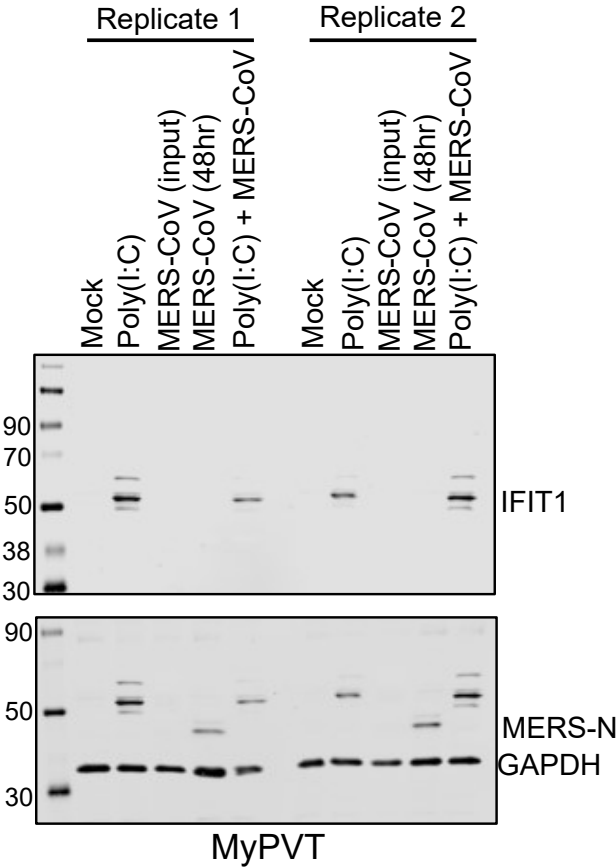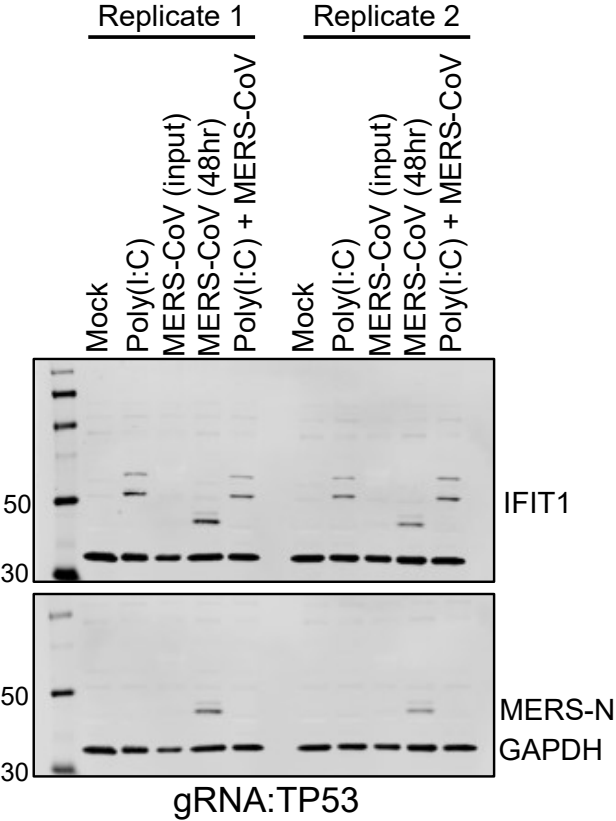

Supplement: S1 Raw images — (PDF) [file pbio.3003098.s005.pdf]
